# Differential expression of immune-regulatory proteins C5AR1, CLEC4A and NLRP3 on peripheral blood mononuclear cells in early-stage non-small cell lung cancer patients

**DOI:** 10.1038/s41598-022-21891-0

**Published:** 2022-11-02

**Authors:** Nussara Pakvisal, Pornrat Kongkavitoon, Chirawadee Sathitruangsak, Nopporn Pornpattanarak, Piyaporn Boonsirikamchai, Pongsakorn Ouwongprayoon, Chatchawit Aporntewan, Poonchavist Chantranuwatana, Apiwat Mutirangura, Chanida Vinayanuwattikun

**Affiliations:** 1 Division of Medical Oncology, Department of Medicine, Faculty of Medicine, Chulalongkorn University and The King Chulalongkorn Memorial Hospital, Bangkok, 10330 Thailand; 2 Holistic Center for Cancer Study and Care (HOCC-PSU) and Medical Oncology Unit, Division of Internal Medicine, Faculty of Medicine, Prince of Songkla University, Hat Yai, 90110 Songkhla Thailand; 3 Department of Surgery, Faculty of Medicine, Chulalongkorn University and the King Chulalongkorn Memorial Hospital, Bangkok, 10330 Thailand; 4 Department of Radiology, Faculty of Medicine, Chulalongkorn University and The King Chulalongkorn Memorial Hospital, Bangkok, 10330 Thailand; 5 Department of Mathematics and Computer Science & Omics Sciences and Bioinformatics Center, Faculty of Science, Chulalongkorn University, Bangkok, 10330 Thailand; 6 Department of Pathology, Faculty of Medicine, Chulalongkorn University and the King Chulalongkorn Memorial Hospital, Bangkok, 10330 Thailand; 7 Center of Excellence in Molecular Genetics of Cancer and Human Diseases, Department of Anatomy, Faculty of Medicine, Chulalongkorn University, Bangkok, 10330 Thailand

**Keywords:** Cancer, Molecular biology, Biomarkers, Oncology

## Abstract

Changes in gene expression profiling of peripheral blood mononuclear cells (PBMC) appear to represent the host’s response to the cancer cells via paracrine signaling. We speculated that protein expression on circulating T-lymphocytes represent T-lymphocyte trafficking before infiltration into the tumor microenvironment. The possibility of using protein expression on circulating T-lymphocytes as a biomarker to discriminate early-stage non-small cell lung cancer (NSCLC) was explored. Four independent PBMC gene expression microarray datasets (GSE12771, GSE13255, GSE20189 and GSE3934) were analyzed. We selected C5AR1, CLEC4A and NLRP3 based on their significant protein expression in tumor-infiltrating lymphocytes, but not in normal lymphoid tissue. A validation study using automated flow cytometry was conducted in 141 study participants including 76 treatment-naive early-stage non-small cell lung cancer patients (NSCLC), 12 individuals with non-malignant pulmonary diseases, and 53 healthy individuals. Median ratios of C5AR1, CLEC4A and NLRP3 specific antibody staining to CD3 positive cells in early-stage NSCLC patients compared to healthy controls were 0.014 [0–0.37] vs. 0.01 [0–0.07, *p* = 0.13], 0.03 [0–0.87] vs. 0.02 [0–0.13, *p* = 0.10] and 0.19 [0–0.60] vs. 0.09 [0.02–0.31, *p* < 0.0001], respectively. Median fluorescence intensity (MFI) of CD3^+^C5AR1^+^, CD3^+^CLEC4A^+^ and CD3^+^NLRP3^+^ expression in early-stage NSCLC patients compared to healthy volunteers was 185 [64.2–4801] vs. 107.5 [27–229, *p* < 0.0001], 91.2 [42.4–2355] vs. 71.25 [46.2–103, *p* = 0.0005], and 1585 [478–5224] vs. 758.5 [318–1976, *p* < 0.0001], respectively. NLRP3:CD3 ratio, CD3^+^C5AR1^+^, CD3^+^CLEC4A^+^ and CD3^+^NLRP3^+^ MFI were significantly higher in early-stage NSCLC than healthy volunteers with an area under the ROC curve of 0.69–0.76. The CD3^+^NLRP3^+^ MFI provided the most distinguishable expression at 71.5% sensitivity and 70% specificity. Furthermore, CD3^+^NLRP3^+^ MFI potentially discriminated between early-stage NSCLC from malignant-mimic inflammation and infection pulmonary disease. Further validation in various pulmonary inflammatory disease might be warranted. Our proof-of-principle findings strengthen the hypothesis that malignancies generate distinctive protein expression fingerprints on circulating T-lymphocytes.

## Introduction

Lung cancer is the most common cancer and the leading cause of cancer-related deaths worldwide with around 2.2 million patients and an estimated 1.8 million deaths in 2020^1^. The majority of lung cancer patients are diagnosed with advanced stage disease in which curative treatment is not suitable. Despite improvement in therapies, the overall 5-year survival rate for all stages is approximately 21%^2^. Thus, there is a major effort for discovery of screening tools for early-stage disease. The National Lung Screening Trial (NLST) using low-dose helical computed tomography (LDCT) demonstrated a 20% reduction in lung cancer mortality among high-risk individuals, but produced a high rate of false-positive results which often led to unneeded invasive procedures^3^. Exploring complementary noninvasive diagnostic testing may offer better results especially if a blood-based biomarker can be identified.

Peripheral blood mononuclear cells (PBMCs) consist of monocytes, T cells, B cells, granulocytes, and natural killer cells and serve as the immune system’s first line of defense against malignancy^4^. The changes in gene expression profiling in PBMCs appear to represent the host’s response to the cancer cells via paracrine signaling^5^. Several studies have demonstrated that the characterization of gene expression in PBMCs may be useful as a diagnostic test for early detection of various types of cancer^4,6–11^. However, several technical issues limit the use of gene expression on PBMCs in real-life practice. The main challenge is timely sample processing because delayed processing affects the gene expression results in PBMCs^12^. Proteins are less complex and usually correlates to mRNA expression on PBMCs^13^. Given these advantages over gene expression characterization, protein expression on PBMCs may provide a more feasible biomarker that could help determine early cancer status.

In this study, we postulated that the immune-related protein expression of PBMCs from early-stage non-small cell lung cancer (NSCLC) patients would differ from that of healthy controls. Discriminative expression of non-malignant pulmonary disease could also enhance the potential possibility of these blood-based biomarkers. Therefore, specific protein expression on PBMCs from three separate groups of early-stage NSCLC patients, non-malignant pulmonary disease patients, and healthy control were conducted.

## Results

### Demographic characteristics of study participants

We enrolled a total of 141 study participants consisting of 76 treatment-naïve early-stage NSCLC patients, 12 non-malignant pulmonary diseases patients, and 53 healthy individuals. The median age of early-stage NSCLC patients was 67 years [range 41–81]. Fifty percent of patients were female. All patients had ECOG performance of 0–1. Nearly all (98.7%) histology showed adenocarcinoma. Pathological stage I disease accounted for 70%. All patients received curative attempt operations including lobectomy and bilobectomy. The median age for the non-malignant pulmonary diseases and healthy controls were 38 years [range 16–68] and 62 years [range 53–64], respectively. Ninety-five percent of non-malignant pulmonary disease patients presented with pulmonary nodules mimicking lung cancer and underwent operative procedures. A majority (70%) of resected lung tissue histology showed infection and inflammation. Details of patient demographics and disease characteristics at study enrollment are presented in Table 1.

**Table 1:** Demographics and disease characteristics of patients with early-stage NSCLC, non-malignant pulmonary diseases, and healthy controls at study enrollment and after quality control (QC) assessment.

Characteristics	Early NSCLC		Non-malignant pulmonary diseases		Healthy controls	
	At enrollment N = 76 (%)	After QC N = 63 (%)	At enrollment N = 12 (%)	After QC N = 10 (%)	At enrollment N = 53 (%)	After QC N = 44 (%)
**Age, n (%)**						
≥ 60 year	61 (80.3)	49 (77.8)	8 (66.7)	7 (70)	10 (18.9)	9 (20.5)
< 60 year	15 (19.7)	14 (22.2)	4 (33.3)	3 (30)	43 (81.1)	35 (79.5)
**Sex, n (%)**						
Male	38 (50)	29 (46.0)	3 (25)	3 (30)	19 (35.8)	15 (34.1)
Female	38 (50)	34 (54.0)	9 (75)	7 (70)	34 (64.2)	29 (65.9)
**Smoking, n (%)**						
Current	4 (5.3)	4 (6.3)	0	0	3 (5.7)	2 (4.5)
Former	23 (30.3)	19 (30.2)	0	0	2 (3.8)	2 (4.5)
Never	45 (59.2)	36 (57.1)	10 (83.3)	9 (90)	46 (86.8)	38 (86.5)
Unknown	4 (5.3)	4 (6.3)	2 (16.7)	1 (10)	2 (3.8)	2 (4.5)
**Histology malignancy, n (%)**						
Adenocarcinoma	75 (98.7)	62 (98.4)	N/A	N/A	N/A	N/A
Squamous carcinoma	1 (1.3)	1 (1.6)				
**Histology non-malignancy, n (%)**						
Granulomatous inflammation	N/A	N/A	3 (25)	3 (30)	N/A	N/A
Chronic and acute inflammation			5 (41.8)	4 (40)		
Sclerosing pneumocytoma			1 (8.3)	1 (10)		
Benign Calcified nodule			1 (8.3)	1 (10)		
Pulmonary harmatoma			1 (8.3)	1 (10)		
Aneurysm of blood vessel			1 (8.3)	0		
**Staging by AJCC**						
0 (TisN0M0)	4 (5)	4 (6)				
IA	31 (41)	26 (41)	N/A	N/A	N/A	N/A
IB	18 (24)	14 (22)				
IIA	8 (10)	6 (10)				
IIB	6 (8)	6 (10)				
IIIA	7 (9)	5 (8)				
IIIB	2 (3)	2 (3)				

*QC* quality control, *N/A* non-applicable.

### Quality control assessment and storage time effect

We explored individual CD3 T-cell positive expression using flow cytometry detection over 5,000 viable PBMCs as the minimal accepted rate for quality control (QC). There were 44 healthy controls (83%), 10 non-malignant pulmonary diseases (83%) patients, and 63 early-stage NSCLC patients (81%) passing QC. Median collected freezing time of PBMCs to flow cytometry detection was 30.6 months [range 2.3–49.2]. While the storage time of passing QC specimen was shorter in healthy volunteers compared to early-stage NSCLC patients with a median of 10.8 [range 2.35–10.96] vs. 40.6 month [range 28.71–49.16] (*p* < 0.0001), the proportion of passing QC was consistent between groups. There was no significant difference in freezing time of PBMCs for passing QC specimen in early-stage NSCLCs and non-malignant pulmonary diseases. Median storage time of PBMCs from non-malignant pulmonary diseases was 31.7 [range 29.61–48.45] months (*p* = 0.08). We concluded that long-term storage in − 196 °C did not affect the quality of protein detection.

### Candidate PBMCs protein expression discovery

We discover the potentially different protein expressions on PBMC in early-stage NSCLC patients compared to healthy controls. The datasets analyzed during the current study are available in the Gene Expression Omnibus repository (available at https://www.ncbi.nlm.nih.gov/geo/) including GSE12771^10^, GSE13255^7^, GSE20189^14^, and GSE39345^15^. Demographic characteristics of patients from 4 gene expression datasets are shown in Table S1. Candidate up- and down-regulated gene expressions from these four independent microarray experiments were identified by using CU-DREAM (Connection Up- and Down-Regulation Expression Analysis of Microarrays)^16^. 1885 significant gene expressions with *p*-values and odd ratios > 1 which indicated a strong association between the two independents studies were retrieved (Table S2). Overlapping candidate gene expressions from each gene expression microarray dataset are illustrated (Fig. S1). Overlapping significant up-regulated genes at least 3 datasets were retrieved for biological functions using the PANTHER (Protein ANalysis THrough Evolutionary Relationships) classification system (available at http://www.pantherdb.org)^17^ (Fig. S2). Seventy-five genes with immune system processes were identified and then mapped with pathology-based protein expression profiling in the Human Protein Atlas (available at https://www.proteinatlas.org)^18^ (Table S3). Three significant up-regulated genes which had the presence of antibody-specific, immunohistochemistry-based protein expression on tumor-infiltrating lymphocytes (TILs) in tumor specimen, but not in normal lymphoid tissue, were selected including CLEC4A, C5AR1, NLRP3. Details of selected gene ontology in homo sapiens based on the PANTHER are summarized in Table S4.

### Protein expression on PBMCs in early-stage lung cancer patients and healthy controls

**Figure 1:**
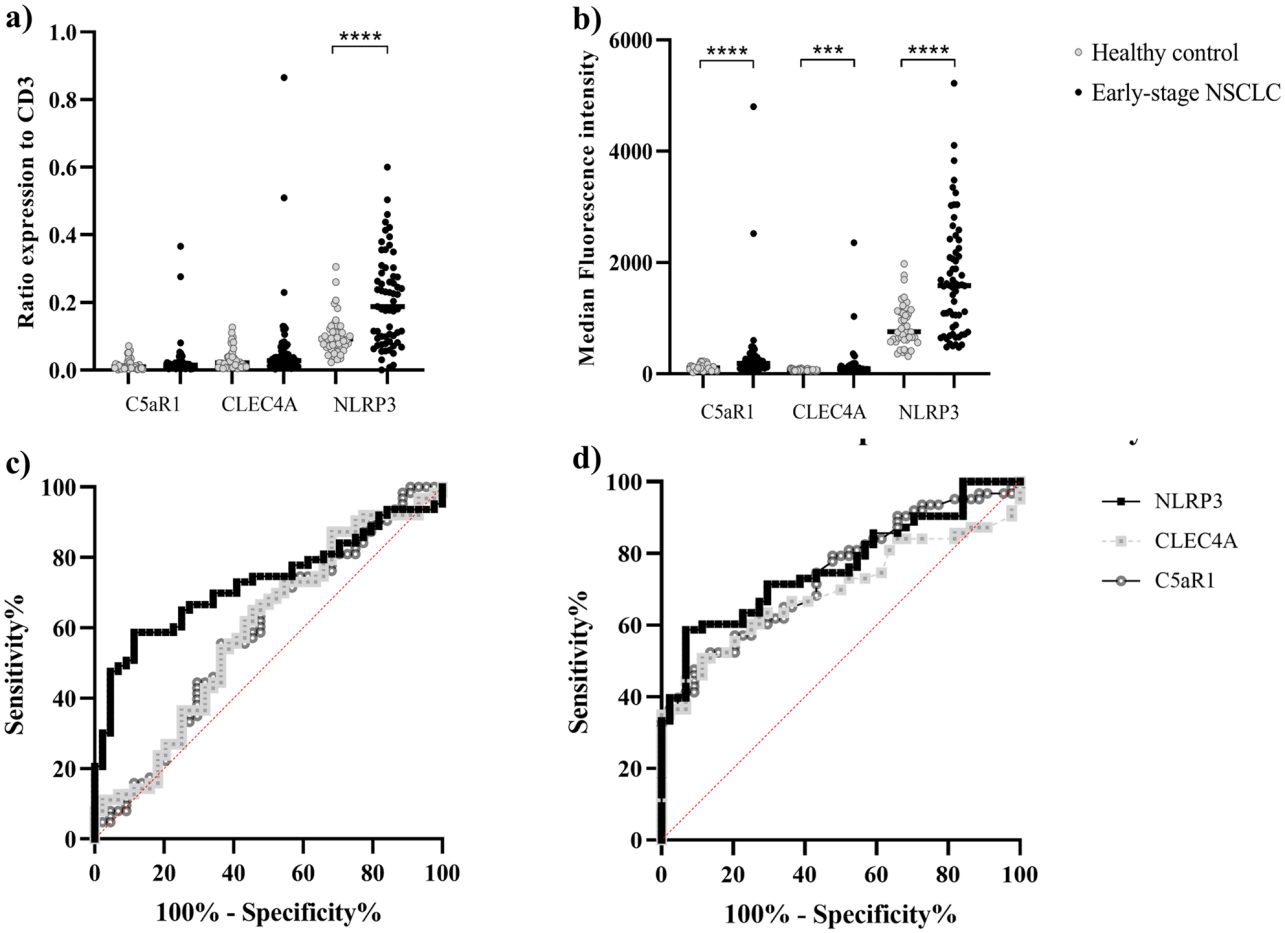
Represented the ratios (**a**), median fluorescence intensity (MFI) (**b**) and adjusted expression (**c**) of C5AR1, CLEC4A and NLRP3 to CD3^+^ lymphocytes between healthy controls (n = 44) and early-stage NSCLC patients (n = 63). The ROC curve of C5AR1, CLEC4A and NLRP3 ratio and MFI between healthy controls and early-stage NSCLC patient (**c**,**d**). The CD3^+^NLRP3^+^ MFI provided the most distinguishable between early-stage NSCLC and healthy volunteer at 71.5% sensitivity and 70% specificity.

We first explored the potential of identifying a biomarker in patients with early-stage lung cancer detection compared to healthy controls. The ratio of specific antibody staining to CD3 positive cells was calculated by dividing the percentage of specific antibody staining cells to the percentage of CD3 positive cells. The median ratios of C5AR1, CLEC4A and NLRP3 expression in early-stage NSCLC patients compared to healthy volunteers were 0.014 [range 0–0.37] vs. 0.01 [range 0–0.07, *p* = 0.13], 0.03 [range 0–0.87] vs. 0.02 [range 0–0.13, *p* = 0.10] and 0.19 [range 0–0.6] vs. 0.09 [range 0.02–0.31, *p* < 0.0001], respectively (Fig. 1a, Table 2). In addition to the number of positive staining cells, flow cytometry also enabled us to determine fluorescence intensity on the cell surface of each specific antibody-stained cell. The median fluorescence intensity (MFI) of CD3^+^C5AR1^+^, CD3^+^CLEC4A^+^ and CD3^+^NLRP3^+^ expressions in early-stage NSCLC patients compared to healthy volunteers were 185 [range 64.2–480] vs. 107.5 [range 27–229, *p* < 0.0001], 91.2 [range 42.4–2355] vs. 71.2 [range 46.2–103, *p* = 0.0005] and 1585 [range 478–5224] vs. 758.5 [range 318–1976, *p* < 0.0001], respectively (Fig. 1b, Table 2). Adjusted fluorescence intensity for specific protein expressions measured the fluorescence intensity if the ratio of specific proteins to CD3 positive cells equaled 1. It was calculated from the ratio of specific antibody staining to CD3 positive cells multiplied by MFI. Median adjusted fluorescence intensity of CD3^+^C5AR1^+^, CD3^+^CLEC4A^+^ and CD3^+^NLRP3^+^ cells in the early-stage NSCLC compared to healthy volunteers were 2.80 [range 0.63–1758.44] vs. 1.01 [range 0.03–15.21, *p* = 0.007], 2.49 [range 0.15–2037.22] vs. 1.39 [range 0.27–12.97, *p* = 0.02], and 286.79 [range 0.41–3138.03] vs. 64.67 [range 11.09–517.74, *p* < 0.0001], respectively.

**Table 2:** Results of C5AR1, CLEC4A and NLRP3 protein expression of circulating T- lymphocyte in study participants.

	Total (n = 117)	Early-stage NSCLC (n = 63)	Non-malignant pulmonary disease (n = 10)	Healthy control (n = 44)
**C5AR1 expression**				
Ratio^#^ [range]	0.01 [0.00–0.37]	0.014 [0.00–0.37]	0.01 [0.00–0.02]	0.01 [0.00–0.07]
Median fluorescence intensity [range]	138.00 [44.90–4801.00]	185.00 [64.20–4801.00]	177.50 [44.90–263.00]	107.50 [27.00–229.00]
Adjusted expression [range]	1.63 [0.03–1758.44]	2.80 [0.63–1758.44]	2.14 [0.21–3.58]	1.01 [0.03–15.21]
**CLEC4A expression**				
Ratio [range]	0.03 [0.00–0.87]	0.03 [0.00–0.87]	0.02 [0.01–0.08]	0.02 [0.00–0.13]
Median fluorescence intensity [range]	77.10 [42.40–2355.00]	91.20 [42.40–2355.00]	84.80 [55.20–161.00]	71.25 [46.20–103.00]
Adjusted expression [range]	2.06 [0.15–2037.22]	2.49 [0.15–2037.22]	2.30 [0.77–12.11]	1.39 [0.27–12.97]
**NLRP3 expression-**				
Ratio [range]	0.11 [0.00–0.60]	0.19 [0.00–0.60]	0.13 [0.07–0.22]	0.09 [0.02–0.31]
Median fluorescence intensity [range]	1067.00 [318.00–5224.00]	1585.00 [478.00–5224.00]	899.00 [354.00–1888.00]	758.50 [318.00–1976.00]
Adjusted expression [range]	126.72 [0.41–3138.03]	286.79 [0.41–3138.03]	114.39 [24.32–413.16]	64.67 [11.09–517.74]

^**#**^Ratio calculated by percentage of positive cells in each specific antibody to CD3^+^ cells.

*Adjusted expression calculated by the ratio of each specific antibody multiply by the average expression.

Fluorescence intensity of C5AR1, CLEC4A and NLRP3 expression on CD3 positive in early-stage NSCLC patients were significantly higher than healthy controls. These results suggest that either MFI or adjusted C5AR1, CLEC4A and NLRP3 protein expression on the circulating T-lymphocytes exhibit changes in the presence of cancer. The ratio of NLRP3 expression on CD3 lymphocytes was also significantly higher in early-stage NSCLC patients compared to healthy controls. These results show the feasibility of using either the NLRP3 ratio or MFI in CD3 positive cells for distinguishing between the early-stage NSCLC and healthy controls.

### Protein expression on PBMC as a potential candidate to discriminate early-stage non-small cell lung cancer patients from healthy controls

For the clinical use of a potential biomarker to discriminate early-stage NSCLC patients from healthy controls, the ratio and MFI cutoff of significant specific antibody staining expressed on CD3^+^ cells were calculated. An NLRP3 ratio of more than 0.12 was found to significantly discriminate early-stage NSCLC patients from healthy volunteers with an area under the ROC curve of 0.72 (*p* < 0.0001). This cutoff provided 60% sensitivity and 75% specificity (Fig. 1c). CD3^+^C5AR1^+^, CD3^+^CLEC4A^+^ and CD3^+^NLRP3^+^ MFI also significantly discriminated between early-stage NSCLC patients and healthy volunteers with an area under the ROC curve of 0.74 (*p* < 0.0001), 0.69 (*p* = 0.0006) and 0.76 (*p* < 0.0001) respectively (Fig. 1d). The CD3^+^C5AR1^+^ MFI of more than 139 units could distinguish early-stage NSCLC patients from healthy controls at 62% sensitivity and 70% specificity. The CD3^+^CLEC4A^+^ MFI of more than 81.5 units could distinguish early-stage NSCLC patients from healthy controls at 60% sensitivity and 75% specificity. Lastly, the CD3^+^NLRP3^+^ MFI of more than 1054 units provided the best sensitivity at 71.5% between early-stage NSCLC patients and healthy controls and 70% specificity.

### Protein expression on PBMC in early-stage lung cancer and non-malignant pulmonary disease

**Figure 2:**
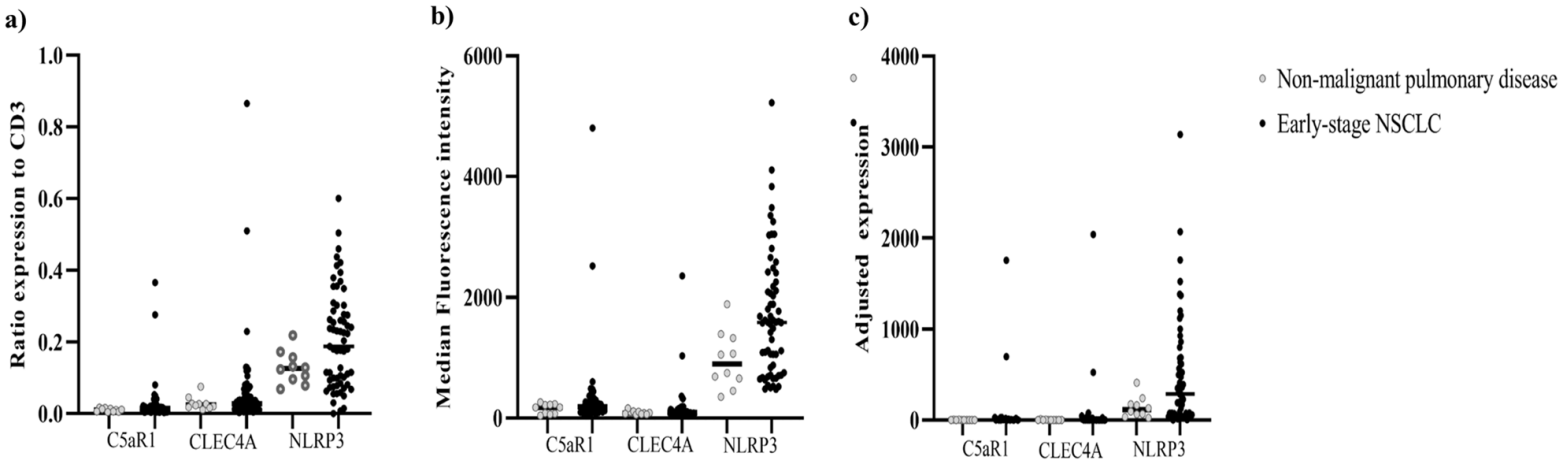
Represented the ratios (**a**), median fluorescence intensity (**b**) and adjusted expression (**c**) of C5AR1, CLEC4A and NLRP3 proteins on CD3^+^ lymphocytes between non-malignant pulmonary diseases (n = 10) and early-stage NSCLC patients (n = 63). Median fluorescence intensity of CD3^+^NLRP3^+^ in early-stage NSCLC patients was 1585 [range 478–5224] which was higher than non-malignant pulmonary diseases, 899 [range 354–1888, *p* = 0.01]. The limited number of patients with non-malignant pulmonary disease in our study impacted the ability to show if PBMCs protein expression as a biomarker could discriminate between these conditions.

An important clinical issue in the discovery of a biomarker is the ability to discriminate non-malignant pulmonary nodules from the early-stage NSCLC patients. We used specific antibodies for staining the candidate proteins on CD3 positive in PBMCs of non-malignant pulmonary diseases compared to early-stage NSCLC patients. The median ratios of C5AR1, CLEC4A and NLRP3 expression in CD3+ of non-malignant pulmonary diseases were 0.01 [range 0–0.02, *p* = 0.12], 0.02 [range 0.01–0.08, *p* = 0.42] and 0.13 [range 0.07–0.22, *p* = 0.14], respectively. Median fluorescence intensity of CD3^+^C5AR1^+^, CD3^+^CLEC4A^+^ and CD3^+^NLRP3^+^ was 177.5 [range 44.90–263, *p* = 0.23], 84.80 [range 55.20–161, *p* = 0.75) and 899 [range 354–1888, *p* = 0.01], respectively. Median adjusted expression of CD3^+^C5AR1^+^, CD3^+^CLEC4A^+^ and CD3^+^NLRP3^+^ was 2.14 [range 0.21–3.58, *p* = 0.14], 2.30 [range 0.77–12.11, *p* = 0.51] and 114.39 [range 24.32–413.16, *p* = 0.07]. The NLRP3 MFI on CD3 positive of early-stage NSCLC patients showed higher expression than non-malignant pulmonary disease whereas C5AR1 and CLEC4A expression levels were not different (Fig. 2a–c). Even though limited number of non-malignant pulmonary disease patients in our study, higher CD3^+^NLRP3^+^ MFI in early-stage NSCLC patients compared to non-malignant pulmonary disease patients probe the possibility of these PBMCs protein expressions as a biomarker to discriminate between those 2 conditions. Further validation in various pulmonary inflammatory disease might warrant.

## Discussion

Previous studies from our group reported paracrine signal-induced PBMC gene upregulation in various cancer types through epigenetic regulation and expression^5,9,19,20^. On this basis, we explored the possibility of using protein expression on circulating T-lymphocytes as a biomarker to discriminate between early-stage NSCLC and healthy individuals by automated flow cytometry analysis. We speculated that protein expression on circulating T-lymphocytes might represent T-lymphocyte trafficking before infiltration into the tumor microenvironment. We chose specific proteins which are related to immune function in either innate and/or adaptive immune processes. NLRP3:CD3 ratio, CD3^+^C5AR1^+^, CD3^+^CLEC4A^+^ and CD3^+^NLRP3^+^ MFI were significantly higher in early-stage NSCLC compared to healthy volunteers with an area under the ROC curve of 0.69–0.76. The CD3^+^NLRP3^+^ MFI provided the highest discriminatory values at 71.5% sensitivity and 70% specificity. Our proof-of-principle findings strengthen the hypothesis that malignancies generate distinctive protein expression fingerprints on circulating T-lymphocytes. Mechanisms of the 3 chosen markers are detailed below.

C5AR1 (complement component 5a receptor 1) is a G protein-coupled receptor for C5a. It functions as a complement receptor and modulates inflammatory responses via chemokine and cytokine signaling pathways. C5AR1 signaling also contributes to the promotion of tumor growth by suppressing the adaptive immune response against tumor antigens and recruits myeloid-derived suppressor cells (MDSCs) into tumor cells^21,22^. A study to evaluate C5AR1 expression by immunohistochemistry in neoplastic and non-neoplastic human tissue sample demonstrated that C5AR1 was strongly expressed by different types of immune cells including monocytes, macrophages, eosinophil, neutrophil and mast cell, but negative staining in T and B lymphocyte^23^, consistent with The Human Protein Atlas database (Fig. S3). Furthermore, C5AR1 expression in cancer cells promotes cancer invasion through motility activation and matrix metalloproteinases^24^. Both these data and our present findings support C5AR1 as a primary mechanism in non-small cell lung cancer.

CLEC4A (C-type lectin domain family 4-member a) or dendritic immunoreceptor (DCIR) have been reported as an immune suppressor of dendritic cells which play a crucial role in the adaptive immune response. CLEC4A expresses on various immune cells depending on the stage of maturation^25^. One study described the role of CLEC4A down-regulation via small hairpin RNA inhibiting tumor progression in animal models^26^. In human tumorigenesis, a previous study explored the role of CLECs and their potential association with reshaping the immune system in the development of hepatocellular carcinoma. The result demonstrated that hepatocellular tissues had significantly higher mRNA levels of CLEC4A and CLEC4L compared to normal liver tissues^27^. CLEC4A is believed to be a biomarker involved in carcinogenesis with further research in NSCLC needed.

NLRP3 (NOD-, LRR- and pyrin domain-containing 3) inflammasome complex plays a role in innate immune signaling. Aberrant NLRP3 are involved in various inflammatory conditions, infection immune responses, and tumorigenesis including lung cancer^28,29^. Several previous studies demonstrated that NLRP3 inflammasome regulates lung cancer proliferation and metastasis through a variety of mechanisms^30–33^ including promoting phosphorylation of Akt, ERK1/2, and CREB, enhancing Snail expression, decreasing E-cadherin expression^30^, and increasing inflammatory cytokine secretion such as interleukin-1 β^32–34^. Our results supported this concept. The ratio of NLRP3 protein expression on CD3 positive cells and MFI in PBMCs in early-stage NSCLC patients had significantly higher levels compared to healthy individuals.

To the best of our knowledge, this is the first study to explore specific protein expressions on PBMCs by automated flow cytometry in early-stage NSCLC. Many different methods were used to measure protein expression. Several limitations of immunohistochemistry have been reported such as various levels of expression being affected by dilution of antibody, diverse clonal of specific antibodies, and interpretation inconsistencies by pathologists. Our group also reported the results of using immunofluorescence to detect particular protein expressions on lymphocytes in early breast cancer detection^35^. While immunofluorescence assay requires laborious hands-on effort to complete, flow cytometry can determine the target particles of a whole cell presentation in various graphic features and can measure large numbers of analyzed cells (up to 300,000 cells per second). Flow cytometry is a high throughput assay which measures fluorescence signals through a laser source. Gating techniques allow for the simultaneously measurement of both intracellular expression and surface protein expression at a single cell level^36^. It can determine both the intrinsic size and the internal complexity of a cell including the extrinsic characteristics such as specific protein expression. Standardized quantitative flow cytometry using median fluorescence intensity unit has had great impact in the research field and the clinic^37^. Automate flow cytometry is a promising tool for the profiling of both cell surface proteins and intracellular protein signaling from peripheral blood samples in clinical application. However, our study has some limitations. First, C5AR1, CLEC4A and NLRP3 expression was not explored in other subsets of immune cells which prohibit comparative expression to CD3+ T-lymphocyte. Second, the results of this study could not serve the explanation of pathophysiological relevance of diverse expression of C5AR1, CLEC4A and NLRP3 in CD3+ T-lymphocyte amount cancer patient and healthy individuals.

## Methods

### Study participants

All subjects in this study were enrolled in The King Chulalongkorn Memorial Hospital and signed a statement of informed consent. This study was approved by the Ethics Committee of the Faculty of Medicine, Chulalongkorn University, Bangkok, Thailand (IRB 211/61). All experimental methods were carried out in accordance with the approved guidelines and regulations. This trial was registered with clinicaltrials.in.th, # TCTR20190508003. Recruited case studies in a prospective manner were classified into three groups as following: (1) 76 patients with early-stage non-small cell lung cancer (NSCLC), (2) 12 patients with non-malignant pulmonary disease, and (3) 53 healthy volunteers with no history of malignant disease and normal chest radiography within 6 months at enrollment. We defined NSCLC staging system based on pathologic results in accordance with either the seventh edition or the eighth edition of the American Joint Committee on Cancer (AJCC) Cancer Staging System at the time of diagnosis^38^. The exclusion criterion were prior systemic therapies including cytotoxic chemotherapy, small molecule targeted therapy, immunotherapy, history of another malignancy within 5 years before enrollment, and receiving corticosteroids or any immunosuppressive agents. All patients were >  = 18 years. Demographic characteristics of individual patients are presented in Table 1.

### Blood collection and preparation

Whole blood samples were collected in EDTA containers from all participants. Peripheral mononuclear cells (PBMC) were isolated using Ficoll–Paque™ PLUS (GE Healthcare Life Sciences, Canada) as previously described^39^. Briefly, blood samples were centrifuged at 1600×*g* at 16 °C for 10 min. After plasma removal, the specimen was diluted (1:1) with phosphate buffered saline (PBS) solution. The diluted specimen was then gently layered on top of the ficoll in a new conical tube and centrifuged at 2000 rpm for 30 min. The interface between the diluted specimen and ficoll layers was collected and then washed twice with 10 ml of PBS solution. Specimens were kept in 5% DMSO mixed with culture media at − 196 °C until further analyzed.

### Staining process and flow cytometry analysis

The staining protocol was adapted from Junqueira et al.^22^ assessment to LSRII flow cytometry. In brief, frozen PBMCs were thawed with 10% FBS (Fetal Bovine Serum) in RPMI (Roswell Park Memorial Institute) 1640 and washed twice with 3% FBS in 1X PBS (Phosphate Buffered Saline). For the extracellular staining, the pellets were resuspended with 3% FBS in 1X PBS and stained with human CD3 (1 μg APC anti-human CD3 antibody, Biolegend), C5aR (1 μg PE/Cyanine7 anti-human CD88 antibody, Biolegend), CLEC4A (1 μg Alexa Fluor® 488 anti-human DCIR/CLEC4A antibody, R&D systems), and cell viability (1 μg 7-AAD Viability Staining Solution, Biolegend) at 4 °C for 2 h in darkness. After incubation, these suspensions were centrifuged at 1500 g and washed three times with 3% FBS in 1X PBS. These pellets were fixed with 4% paraformaldehyde (PFA) at room temperature for 10 min and washed triplicate with 3% FBS in PBS. Fixed PBMCs were permeabilized with 0.1% Triton X-100 in 1X PBS for 10 min and washed twice with 3% FBS in 1X PBS. For the intracellular staining, cells were resuspended with 3% FBS in 1X PBS and stained with human NLRP3 (1 μg PE anti-human/mouse NLRP3/NALP3 antibody, R&D systems) and incubated at 4 °C for 2 h in a dark place. In the final step, these pellets were washed three times and then resuspended in 1% PFA in 1X PBS. The samples were kept at 4 °C until flow cytometry analysis. In cases of the isotype control, PE/Cyanine7 mouse IgG2a, Alexa Fluor 488 mouse IgG1 and PE rat IgG2a from Biolegend® were used instead of human C5AR1, CLEC4A and NLRP3 antibodies. These results were analyzed by using FlowJo Version 10 for discriminating cell death and using viable PBMCs more than 5,000 CD3^+^ as the threshold of detection.

For the analytical process, all samples were previously gated only CD3+ lymphocyte cells from other immune cells and debris. We used one of the healthy individual samples for setting the compensation pattern. In the compensation setting, unstained and single-stained fluorescence samples were used as the negative and positive controls. After adjusting the positive area of each fluorescence, we set the cut-off boundary values between one fluorescence signal and the others. (Fig. S4). Finally, this setting compensation was applied for all samples. We used the isotype control as the negative control for non-specific binding. The examples of both the isotype control and sample are shown in Fig. S5.

### Statistical analysis

Statistical parameters used to delineate protein expression on T-lymphocytes were as follows: (1) ratio of specific-antibody positive to CD3^+^ cells, (2) fluorescence intensity of each protein on CD3^+^ cells, and (3) adjusted specific protein expression on CD3^+^ by the ratio of each protein to CD3 multiplied by the fluorescence intensity. Mann–Whitney U test was used to assess differences in non-parametric continuous ratios of expression and adjusted specific proteins to CD3 positive between groups. Receiver operative characteristic (ROC) curve analysis was used to compute sensitivity and specificity of protein expressions as a potential diagnostic marker for early-stage NSCLC compared to healthy controls. The two-sided test *p-*values < 0.01 were considered significant. All the statistical analyses were performed with the use of GraphPad Prism, version 9.

## Supplementary Information

Supplementary Information 1.

Supplementary Information 2.

## Data Availability

The datasets analyzed during the current study are available in the Gene Expression Omnibus repository (available at https://www.ncbi.nlm.nih.gov/geo/) including GSE12771^10^, GSE13255^7^, GSE20189^14^, and GSE39345^15^.

## Abbreviations

AJCC: American Joint Committee on Cancer
C5AR1: Complement component 5a receptor 1
CD: Cluster of differentiation
CLEC4A: C-type lectin domain family 4 member A
CU-DREAM: Connection Up- and Down-Regulation Expression Analysis of Microarrays
FITC: Fluorescein isothiocyanate
MDSCs: Myeloid-derived suppressor cells
NSCLC: Non-small cell lung cancer
PANTHER: Protein ANalysis THrough Evolutionary Relationships classification system
TILs: Tumor-infiltrating lymphocytes
