# Supplementary material for: Differential expression of immune-regulatory proteins C5AR1, CLEC4A and NLRP3 on peripheral blood mononuclear cells in early-stage non-small cell lung cancer patients
Source: Sci Rep. 2022 Nov 2;12:18439. doi: 10.1038/s41598-022-21891-0 (PMC9630369; doi:10.1038/s41598-022-21891-0)
Supplement: Supplementary file 2 — Supplementary Information 2. [file 41598_2022_21891_MOESM2_ESM.docx]

**Differential Expression of Immune-Regulatory Protein C5AR1, NLRP3 and CLEC4A on Peripheral Blood Mononuclear Cells in Early-stage Non-Small Cell Lung Cancer Patients**

Nussara Pakvisal MD^1^*, Pornrat Kongkavitoon PhD^1^*, Chirawadee Sathitruangsak MD^1,2^, Nopporn Pornpatrananrak MD^3^, Piyaporn Boonsirikamchai MD^4^, Pongsakorn Ouwongprayoon MD^4^, Chatchawit Aporntewan PhD^5^, Poonchavist Chantranuwat MD^6^, Apiwat Mutirangura, MD, PhD^7^, Chanida Vinayanuwattikun MD, Ph.D^1^

*represented co-first author

**Author’s affiliations**

^1^Division of Medical Oncology, Department of Medicine, Faculty of Medicine, Chulalongkorn University and The King Chulalongkorn Memorial Hospital, Bangkok, 10330, Thailand

^2^Holistic Center for Cancer Study and Care (HOCC-PSU) and Division of Medical Oncology, Department of Internal Medicine, Faculty of Medicine, Prince of Songkla University, Hat Yai, Songkhla, 90110, Thailand

^3^Department of Surgery, Faculty of Medicine, Chulalongkorn University and the King Chulalongkorn Memorial Hospital, Bangkok, 10330, Thailand

^4^Department of Radiology, Faculty of Medicine, Chulalongkorn University and The King Chulalongkorn Memorial Hospital, Bangkok, 10330, Thailand

^5^Department of Mathematics and Computer Science & Omics Science and Bioinformatics Center, Faculty of Science, Chulalongkorn University, Bangkok 10330, Thailand

^6^Department of Pathology, Faculty of Medicine, Chulalongkorn University and the King Chulalongkorn Memorial Hospital, Bangkok, 10330, Thailand

^7^Center for Excellence in Molecular Genetics of Cancer and Human Diseases, Department of Anatomy, Faculty of Medicine, Chulalongkorn University, Bangkok 10330, Thailand

**Corresponding author:** Chanida Vinayanuwattikun MD., PhD. Division of Medical Oncology, Department of Medicine, Faculty of Medicine, Chulalongkorn University and The King Chulalongkorn Memorial Hospital, 256 Rama IV Rd, Pathumwan, Bangkok 10330, Thailand

E-mail : [Chanida.Vi@chula.ac.th](mailto:Chanida.Vi@chula.ac.th)

**Supplementary Figure**

**Figure S1:** Venn diagram of significant gene expression from four datasets, including GSE12771, GSE13255, GSE20189 and GSE39345. (This figure was generated by R package “VennDiagram” https://CRAN.R-project.org/package=VennDiagram)


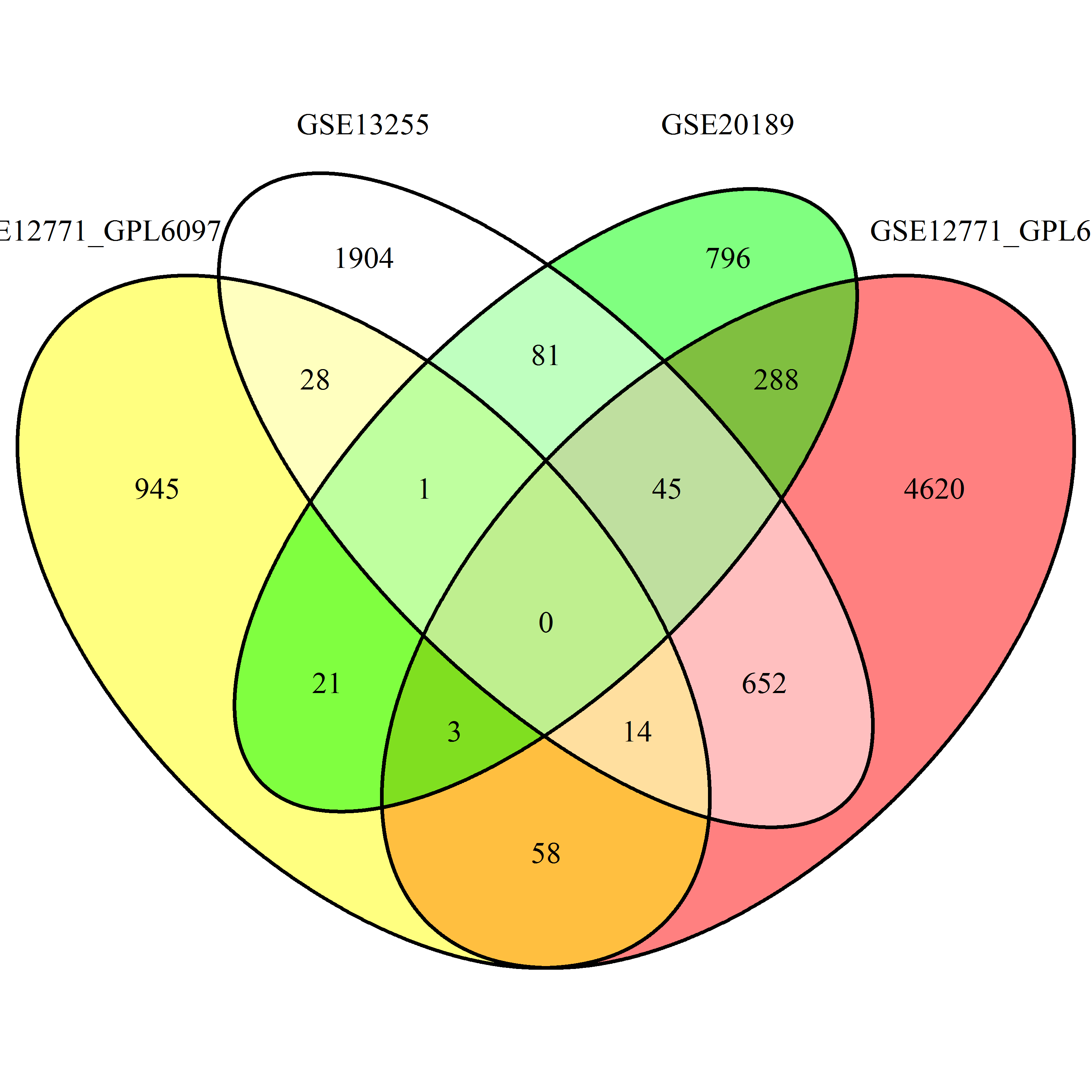


**Figure S2** Overlapping significant up-regulation genes. More than 3 datasets (1807 genes) were retrieved for biological functions using the PANTHER (Protein ANalysis THrough Evolutionary Relationships) classification system (This figure was generated using <http://www.pantherdb.org>)

**
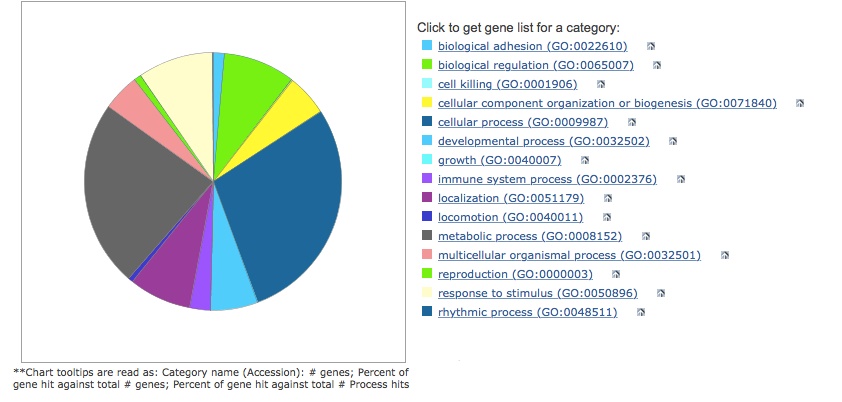
**

**Figure S3** represented selection population of specific antibody with CD3 positive cells. (This figure was generated by FlowJo Version 10. https://www.flowjo.com/learn/flowjoportal)
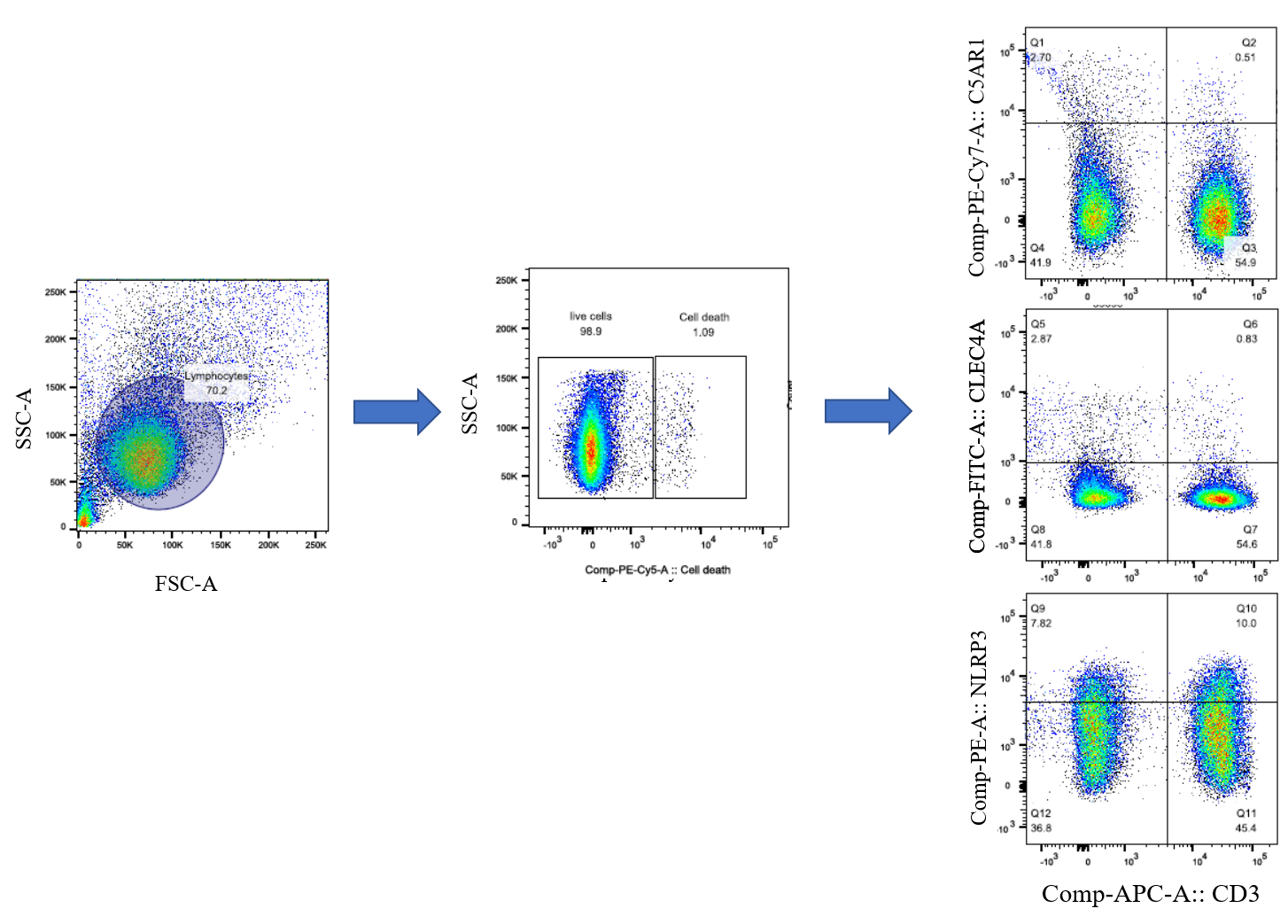


**Figure S4** shows the process of compensation in this study. After gating lymphocytes, each fluorescence was minus by the negatively staining sample and set the positive fluorescence area (a). All fluorochromes were setting the value by opting-out the overlap signals (b). (This figure was generated by FlowJo Version 10. https://www.flowjo.com/learn/flowjoportal)


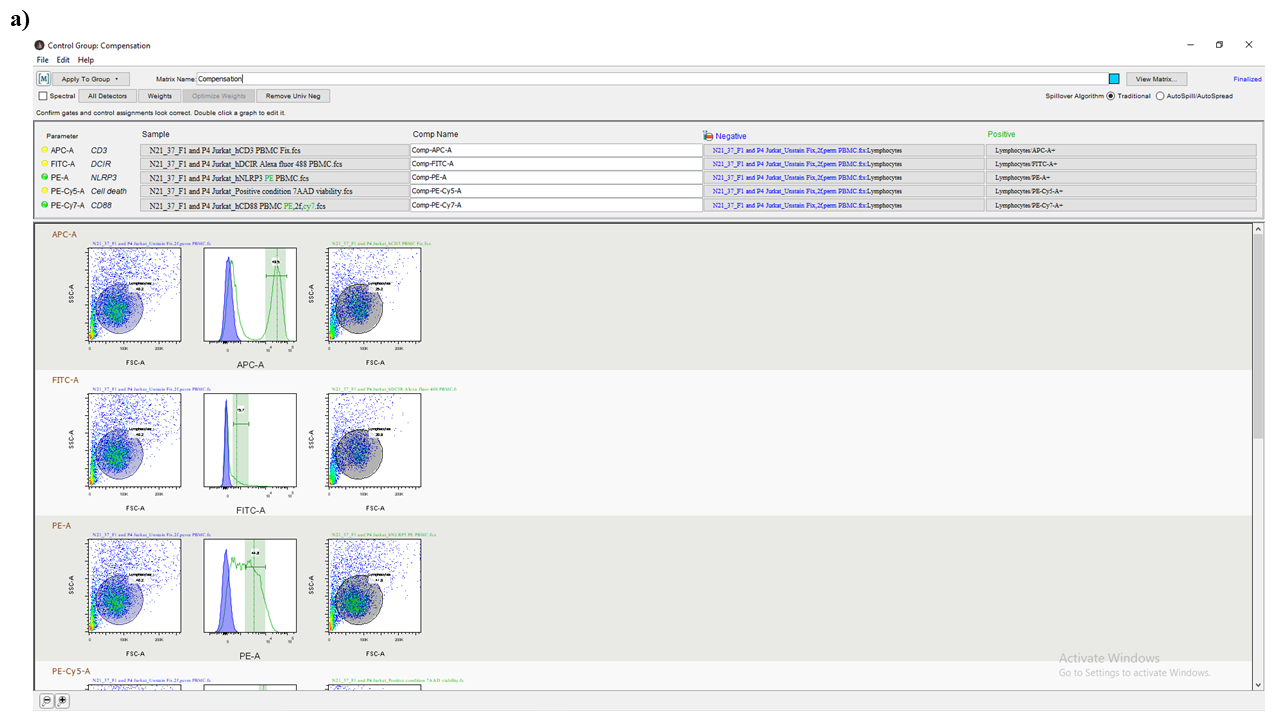

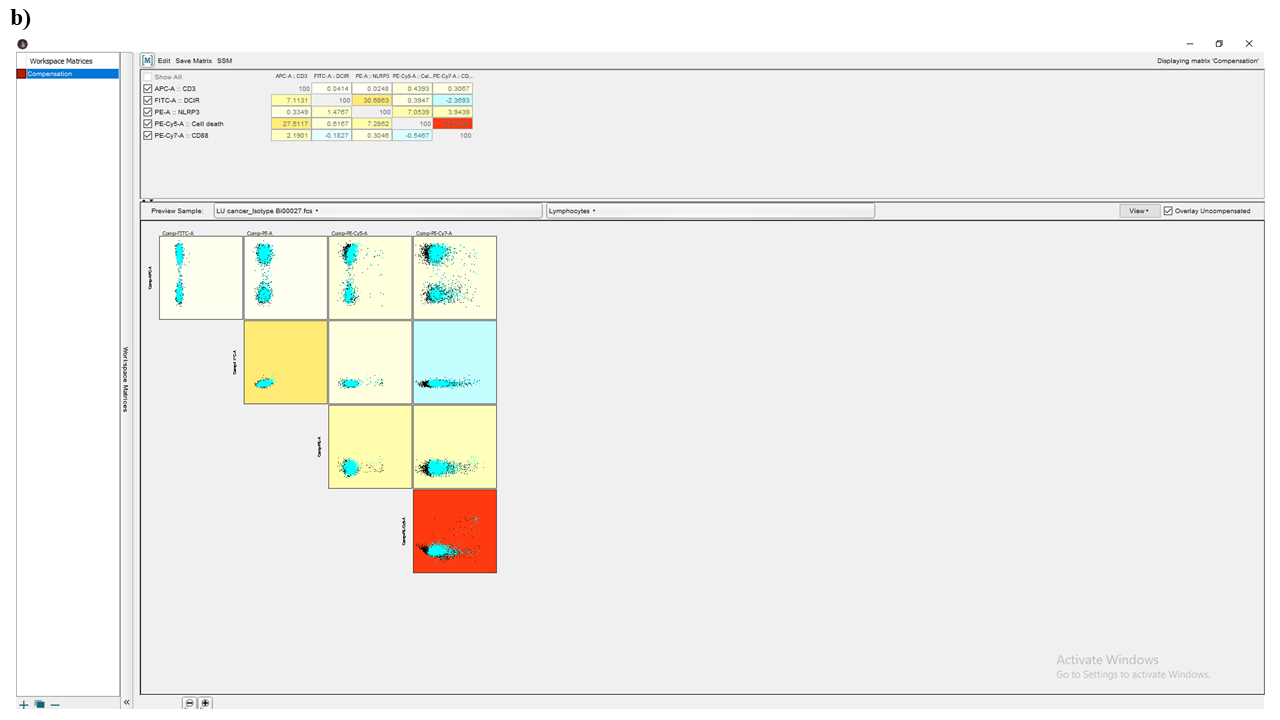


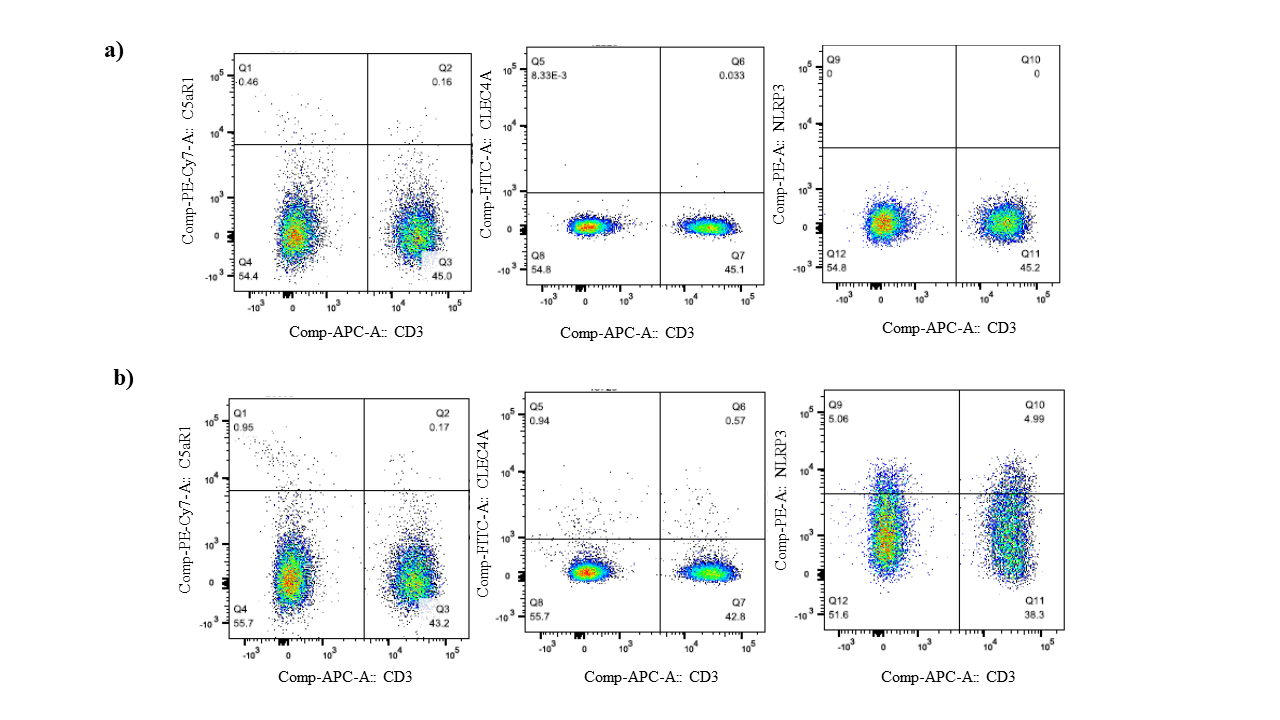
**Figure S5** Example of the isotype control staining (a) and the specific positive staining on CD3^+^ lymphocytes (b). (This figure was generated by FlowJo Version 10. https://www.flowjo.com/learn/flowjoportal)

**Table S4** Gene ontology of *CLEC4A, C5AR1* and *NLRP3* in Homo sapiens species based on the Protein ANalysis THrough Evolutionary Relationships classification system (available at <http://www.pantherdb.org>).

| **Gene** | **PANTHER family** | **PANTHER subfamily** | **Protein class** | **Pathway categories** |
| --- | --- | --- | --- | --- |
| *CLEC4A* | C-type lectin superfamily member | C-type lectin domain family 4 member A | Cell adhesion molecule, immunoglobulin receptor superfamily | N/A |
| *C5AR1* | Chemotactic receptor | C5A anaphylatoxin chemotactic receptor 1 | G-protein coupled receptor | Inflammation mediated by chemokine and cytokine signaling pathway |
| *NLRP3* | Family not named | NOD-, LRR- and pyrin domain-containing 3 | N/A | N/A |

N/A, not applicable
